# A machine learning model on Real World Data for predicting progression to Acute Respiratory Distress Syndrome (ARDS) among COVID-19 patients

**DOI:** 10.1371/journal.pone.0271227

**Published:** 2022-07-28

**Authors:** Nicola Lazzarini, Avgoustinos Filippoupolitis, Pedro Manzione, Hariklia Eleftherohorinou

**Affiliations:** 1 Real World Analytics & AI, IQVIA, Cambridge, United Kingdom; 2 Strategic Analytics & Insights, IQVIA, Saint-Prex, Switzerland; 3 Innovation Ventures & Strategic Partnerships, IQVIA, Athens, Greece; University Medical Center Goettingen, GERMANY

## Abstract

**Introduction:**

Identifying COVID-19 patients that are most likely to progress to a severe infection is crucial for optimizing care management and increasing the likelihood of survival. This study presents a machine learning model that predicts severe cases of COVID-19, defined as the presence of Acute Respiratory Distress Syndrome (ARDS) and highlights the different risk factors that play a significant role in disease progression.

**Methods:**

A cohort composed of 289,351 patients diagnosed with COVID-19 in April 2020 was created using US administrative claims data from Oct 2015 to Jul 2020. For each patient, information about 817 diagnoses, were collected from the medical history ahead of COVID-19 infection. The primary outcome of the study was the presence of ARDS in the 4 months following COVID-19 infection. The study cohort was randomly split into training set used for model development, test set for model evaluation and validation set for real-world performance estimation.

**Results:**

We analyzed three machine learning classifiers to predict the presence of ARDS. Among the algorithms considered, a Gradient Boosting Decision Tree had the highest performance with an AUC of 0.695 (95% CI, 0.679–0.709) and an AUPRC of 0.0730 (95% CI, 0.0676 – 0.0823), showing a 40% performance increase in performance against a baseline classifier. A panel of five clinicians was also used to compare the predictive ability of the model to that of clinical experts. The comparison indicated that our model is on par or outperforms predictions made by the clinicians, both in terms of precision and recall.

**Conclusion:**

This study presents a machine learning model that uses patient claims history to predict ARDS. The risk factors used by the model to perform its predictions have been extensively linked to the severity of the COVID-19 in the specialized literature. The most contributing diagnosis can be easily retrieved in the patient clinical history and can be used for an early screening of infected patients. Overall, the proposed model could be a promising tool to deploy in a healthcare setting to facilitate and optimize the care of COVID-19 patients.

## Introduction

The pandemic caused by the Coronavirus 2019 (COVID-19) disease has distressed the global population and severely strained healthcare systems. Currently, more than 180 million people have been infected by the SARS-CoV-2 and more than 3 million people have already died. Symptoms of COVID-19 vary, but primarily include fever, dry cough, tiredness, headache, loss of smell or taste. According to the Centre for Disease Control and Prevention [1], most of the infected people (81%) develop mild to moderate symptoms. Around 14% develop severe symptoms (dyspnoea, hypoxia, or more than 50% lung involvement on imaging), while 5% suffer critical symptoms (shock, respiratory failure, or multiorgan dysfunction). People affected by severe and critical symptoms often require hospitalisation, ICU and ventilation. It is therefore crucial to identify risk factors that can help to prevent the development of severe symptoms and can aid to predict the patients that could most likely could professional healthcare support.

Machine Learning (ML) is the branch of computer science that creates models and solutions by analysing data, learning, and adapting without following explicit instructions. Different techniques and algorithms exist within the machine learning field: from decision trees where predictions are made following a nested set of tests on attributes, to logistic regression that calculates the probability of events as a linear combination of input features, to boosted algorithms that combine many simple models trained on previous classification errors [2]. Ensemble techniques have also been successfully proposed to combine multiple models together and enhance their overall performance [3]. ML has been proven powerful and effective in many different fields, including healthcare [4]. Over the years, many ML-based solutions of various types have been developed to diagnose diseases, predict severity of infections, estimate likelihood of hospital readmissions, etc. As the COVID-19 pandemic hit globally ML solutions started being proposed, primarily for early detection of the disease or for estimating the severity of the infection. The work presented in this study focuses on the latter. Severity has been defined as inpatient hospitalization by the work of Kenneth et al. [5], uncovering clinical risk factors using an XGBoost classifier. Similarly, more than 500 thousand patients claim data were used to generate an ML model predicting COVID-19 severity, using death as proxy [6]. Hospitalised patients that were transferred to ICU defined the severe group for the predictive models analysed in [7–9]. The same studies also tried to predict severity defined as the need for ventilation, using various ML algorithms to tackle this problem such as XGBoost, Artificial Neural Networks and Random Forest. Electronic Health Records (EHR) data from Mount Sinai Hospital retrospectively collected, were used by Cheng et al. [10] to train a Random Forest classifier that could identify severe patients as admitted to the ICU. Respiratory infections have also been used as a proxy for severity. In [11], a prospective study aimed to estimate a 48-hours prediction of moderate to severe respiratory failure, requiring mechanical ventilation, in hospitalized patients with COVID-19 pneumonia. A total of 198 patients were used to train four predictive models, achieving an accuracy of 84%. In [12], patients from Wuhan and non-Wuhan areas with Acute Respiratory Distress Syndrome (ARDS) were considered severe cases and employed to train and evaluate five different ML algorithms. Using Medicare and Medicaid data, DeCaprio et al. [13] defined severe COVID-19 patients if diagnosed with ARDS, or any of four others closely related respiratory diagnosis. Logistic Regression and XGBoost were subsequentially used to develop three different predictive models that have been validated both internally and externally (using new admission data). A framework based on feature selection and three ML predictive models was developed in [14] to identify who could develop severe illness, specifically ARDS, from patients with mild COVID-19. A machine learning algorithm called “eARDS” was developed to predict early onset of ARDS in an ICU population comprising COVID-19 patients, up to 12-hours before satisfying the Berlin clinical criteria. The model was develop using features extracted from EMR data and obtained an Area Under the ROC Curve (AUROC) of 0.89 [15]. Deep learning was also used to evaluate COVID-19 severity. In [16], the authors developed a combination of a variational autoencoder and a fully connected network to classify patients on different severity levels, defined on the presence of respiratory problems and mortality. Performance appeared to increase together with severity risk. Guidelines from the *Diagnosis and Treatment Protocol for COVID-19 (seventh edition)* were used by Xiong et al. to categorise severe COVID-19 patients and develop a random forest model to predict its presence [17].

In this work, we present an ML model, developed using a large scale nationally relevant integrated dataset, to predict COVID-19 severity, defined as presence of ARDS within four months of the initial infection. In contrast to most of the studies presented so far, that focused on small cohorts and/or with limited patient information, we trained a model using more than 800 diagnosis codes for almost 290,000 patients infected with COVID-19. Collecting information for such a large number of patients guarantees a realistic representation of both severe and non-severe patients, similar to what can be observed in the real world. Hence, the development of a more reliable and precise predictive model, that is quite often not fully achievable when using on smaller cohorts. The model we present was the best performing among three different machine learning algorithms. We evaluated model performance using an unseen test set, to provide an unbiased real-world performance estimation following best practices. Furthermore, we have compared the predictive ability of the model with the predictions provided by a panel of five clinicians. Finally, we have used a state-of-the-art interpretability methodology to identify the most important risk factors for the prediction of ARDS development in COVID-19 patients.

## Materials and methods

### Dataset

The analysis undertaken in this work was based on IQVIA longitudinal Prescription Data (LRx), and Office-Based Practitioners Medical Claims Data (Dx). For the LRx dataset, IQVIA obtains around 4 billion prescription claims per year with history from January 2004, and they provide coverage up to 92% for retail pharmacy prescriptions. Dx data are pre-adjudicated claims collected from office-based physicians and specialists. These data are sourced from CMS-1500 form-based claim transactions, the standard reimbursement form for all non-cash claims. Medical claims data includes patient-level diagnosis and procedures for visits to U.S. office-based individual professionals, ambulatory and general healthcare sites. The medical claims data includes more than 205 million non-identified patients, over 1.7 billion claims and 3 billion service records obtained annually.

### Study cohorts

**Fig 1:**
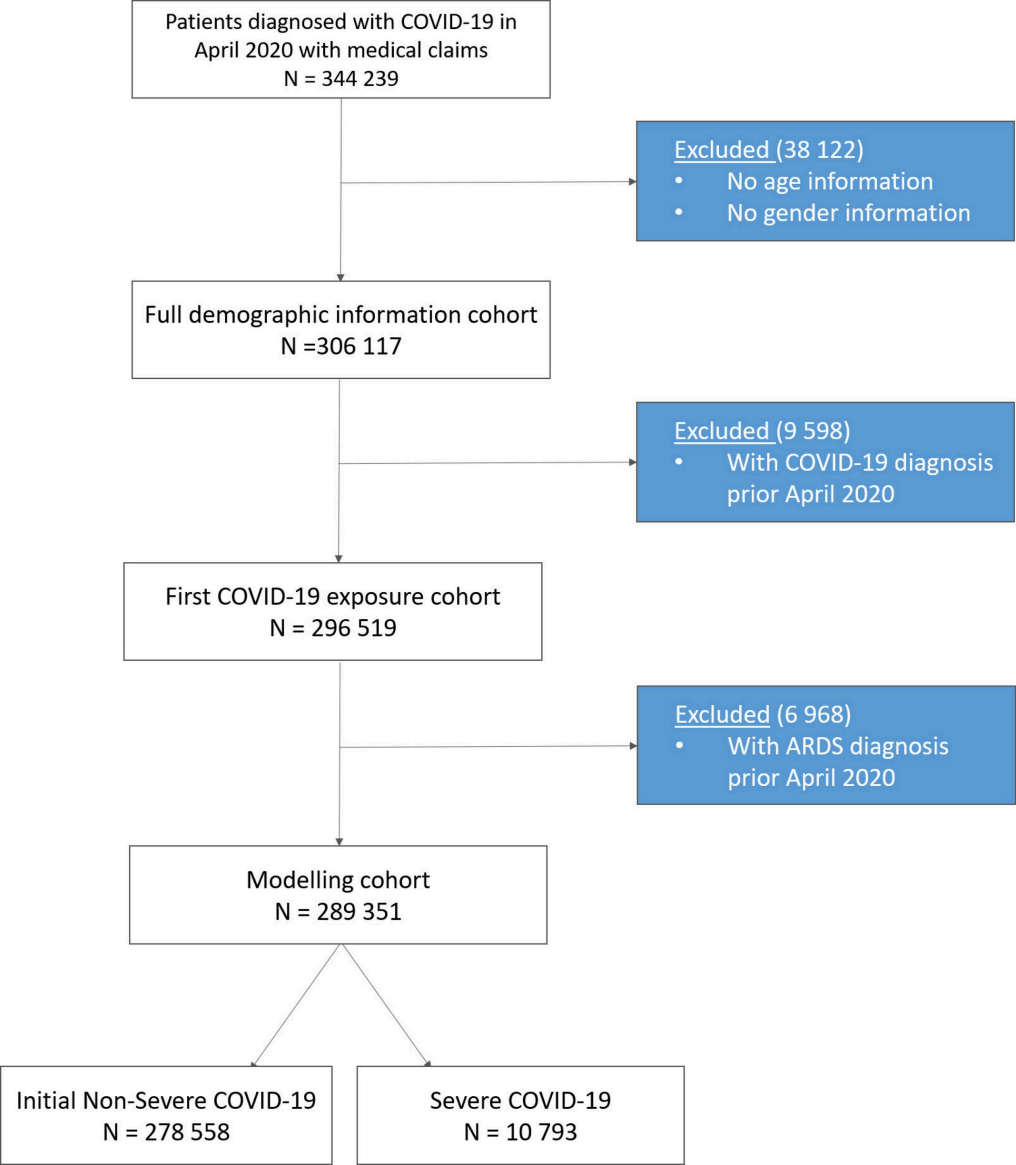
Consort diagram for the study. Consort flow diagram illustrating the rules implemented for selecting patients for modelling cohort.

Patients were included in the initial cohort if they were diagnosed for the first time with COVID-19 (ICD-10 code U07.1) in the month of April 2020. That is, they had no claims with ICD code U07.1 before April 2020. Furthermore, to be included in the initial cohort, the patients should have never been diagnosed with ARDS (ICD-10 code J80) between October 2015 and January 2020. A patient was then labelled as positive (case) if diagnosed with ARDS by the end of July 2020, otherwise the patient was labelled as negative (control). Patients with missing age or gender information were discarded from the analysis. In this study we decided to focus only on patients diagnosed in April 2020 because up to March 2020, the code U07.1 was used for any type of coronavirus infection, not specifically for COVID-19. After all the filters and rules were applied, a total of 289,351 patients were selected for the study: 10,793 had developed severe COVID-19, while 278,558 did not progress into a severe state. The Consort Diagram for the study is shown in Fig 1. This resulted in a ratio of positive-to-negative patients of 1:26. Baseline characteristics of the cohort population are shown in Table 1. We should note that most patients in our cohort are adults, and only 2% of patients are less than 18 years old.

**Table 1:** Baseline characteristics of patients in the study cohort. Patients are defined as “Severe” if diagnosed with Acute Respiratory Distress Syndrome (ARDS) within 4 months from COVID-19 infection. The ten most prevalent comorbidities in the study cohort are shown alongside their respective ICD-10 codes.

	Severe COVID-19 patients	Non-severe COVID-190 patients
**Total patients count**	10,793	278,558
**Gender**		
Male (%)	59.6%	47.3%
Female (%)	40.4%	52.7%
**Age**		
Mean	62.3	53.8
Standard deviation	13.6	17.9
**Essential (primary) hypertension (I10)**	62.8%	46.5%
**Disorders of lipoprotein metabolism and other lipidemias (E78)**	52.8%	40.3%
**Other joint disorder (M25)**	38.7%	36.1%
**Dorsalgia (M54)**	36.6%	34.6%
**Other and unspecified soft tissue disorders (M79)**	38.0%	34.3%
**Abdominal and pelvic pain (R10)**	32.3%	33.3%
**Pain in throat and chest (R07)**	34.3%	30.5%
**Diabetes mellitus (E08)**	45.1%	29.5%
**Abnormalities of breathing (R06)**	38.4%	28.1%
**Malaise and fatigue (R53)**	29.1%	27.5%

We defined as *lookback*, the period from October 2015 (introduction of the ICD-10 code system) to January 2020. The claim history within the lookback was used to construct the feature space of each patient, based on ICD-10 diagnostic codes. That is, the feature set of each sample was representing the comorbidities present in the medical history, up to October 2015. We have forced a gap of a few months between the end of the lookback and the selection period (April 2020) to avoid considering features that could be correlated with COVID-19. Although the infections were diagnosed in April 2020, their symptoms could have easily started weeks earlier and impacted the patient’s history. Using features potentially collected during the COVID-19 infection would have biased the model and would have generated misleading insights.

**Fig 2:**
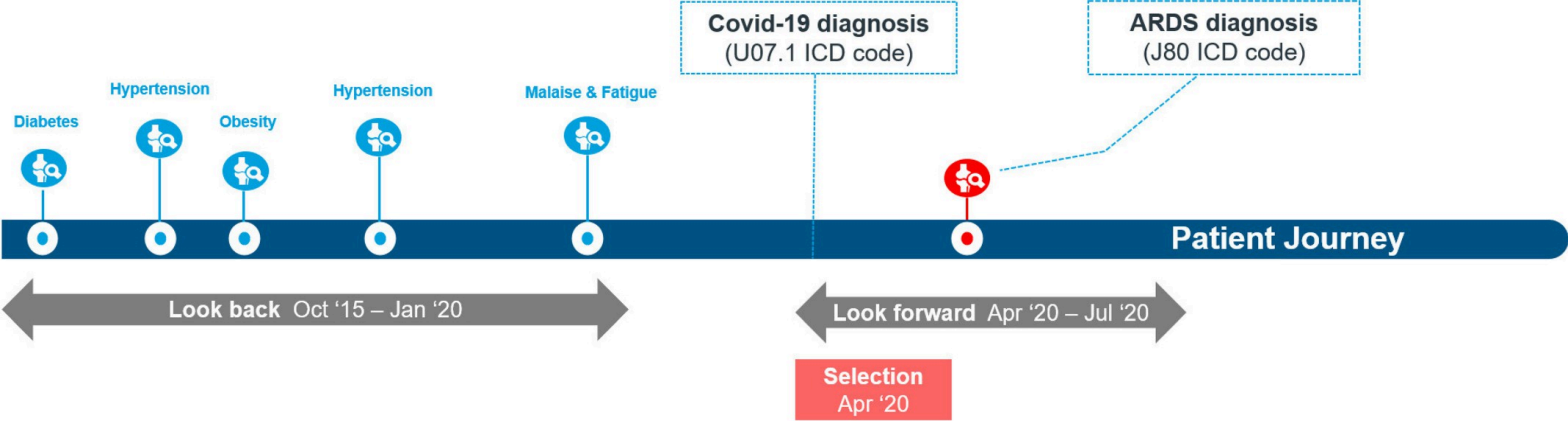
Overview of the cohort definition process. The modelling cohort includes patients diagnosed for the first time with COVID-19 in April 2020. If ARDS is diagnosed by July 2020, the patient is considered a severe case, otherwise non-severe. The period from October 2015 to January 2020 is used to collect diagnosis information for each patient and build the feature set.

A total of 817 boolean comorbidities features were generated for each patient. The value of each feature was set to 1 if the patient had a diagnosis for that disease within the lookback window, otherwise it was set to 0. The complete list of ICD-10 codes used as input space is available as S1 File. Age and gender were also used as input features. The approach used to generate the cohort is illustrated in Fig 2.

### Machine learning modelling

Patients were split into training, validation, and test datasets, respectively defined as 80%, 10% and 10% of the original cohort, using stratified sampling (to maintain the original class ratio in each data split). The training set was used to build and optimize the model, the validation set was used to evaluate different versions of the model, while the test set was used to evaluate its final performance. We maintained the original class distribution of 1:26 in all datasets. Finally, 100 patients were randomly selected from the test set and shared with five clinicians for a predictive performance comparison between the ML model and their expert knowledge.

We evaluated three different machine learning algorithms, widely used to generate predictive models for many complex problems, including healthcare: Logistic Regression [18], Random Forest [19] and LightGBM [20].

Logistic regression is a statistical model that uses a logistic function to model a binary dependent variable (e.g. health status). Random Forest is based on an ensemble learning approach that combines many decision trees, built on different sample sets, to enhance its prediction. Finally, LightGBM is an efficient, accurate and popular machine learning algorithm based on Gradient Boosting Decision Trees (GBDT). The hyper-parameters of a classifier (e.g. number of decision trees, number of learning iterations, etc.) can have a significant impact on its performance, especially when dealing with complex and difficult healthcare problems. Therefore, choosing the right parameter values is crucial to create a good model. Traditionally, hyper-parameters are selected with a grid search, an exhaustive searching through a manually specified subset of values. Instead of grid search, for every classifier, we used Hyperopt [21] a Bayesian optimizer that iteratively evaluates subset of hyper-parameter values and automatically identifies the direction towards moving to improve the results. Hyperopt identified the best set of hyper-parameters that maximized the AUCPR, a metric that summarizes the information of the precision-recall curve in one value, considering both precision and recall at different decision thresholds [22].

### Performance evaluation

The performance of the models was evaluated using both the Receiver Operating Characteristic (ROC) and the Precision-Recall (PR) curve. The ROC curve summarizes the trade-off between the true positive rate and false positive rate for different probability thresholds, while the PR curve summarizes the trade-off between the true positive rate (also known as recall) and the positive predictive value (also known as precision). We also generated additional summary statistics from the two curves by computing the area under the curves. The area under the ROC curve is known as AUC (Area Under the Curve) or C statistic, while the area under the PR curve is referred to as the area under precision recall curve (AUPRC). ROC curves can be misleading when dealing with imbalanced datasets (as occurring in this study). In particular, when the number of negative samples is large, the false positive rate increases and provides a misleading estimate of the model’s performance [23]. Conversely, precision is not affected by the number of negative samples and offers a more reliable performance metric. 95% Confidence Intervals (CI) are calculated, using the bootstrapping method, for both the C statistic and the AUPRC.

### Model interpretability

We used the Shapley Additive Explanations (SHAP) methodology [24–26] to assess the average impact and importance of each feature on the best model’s prediction. A SHAP value is a rigorous mathematical representation of a feature’s contribution to a single prediction generated by the model. A key property of SHAP values is that they are linearly additive. We exploited this property, and we used the mean of the absolute SHAP values of a feature across all patients in the cohort as a measure of this feature’s average impact on model prediction.

## Results

### Model performance

**Fig 3:**
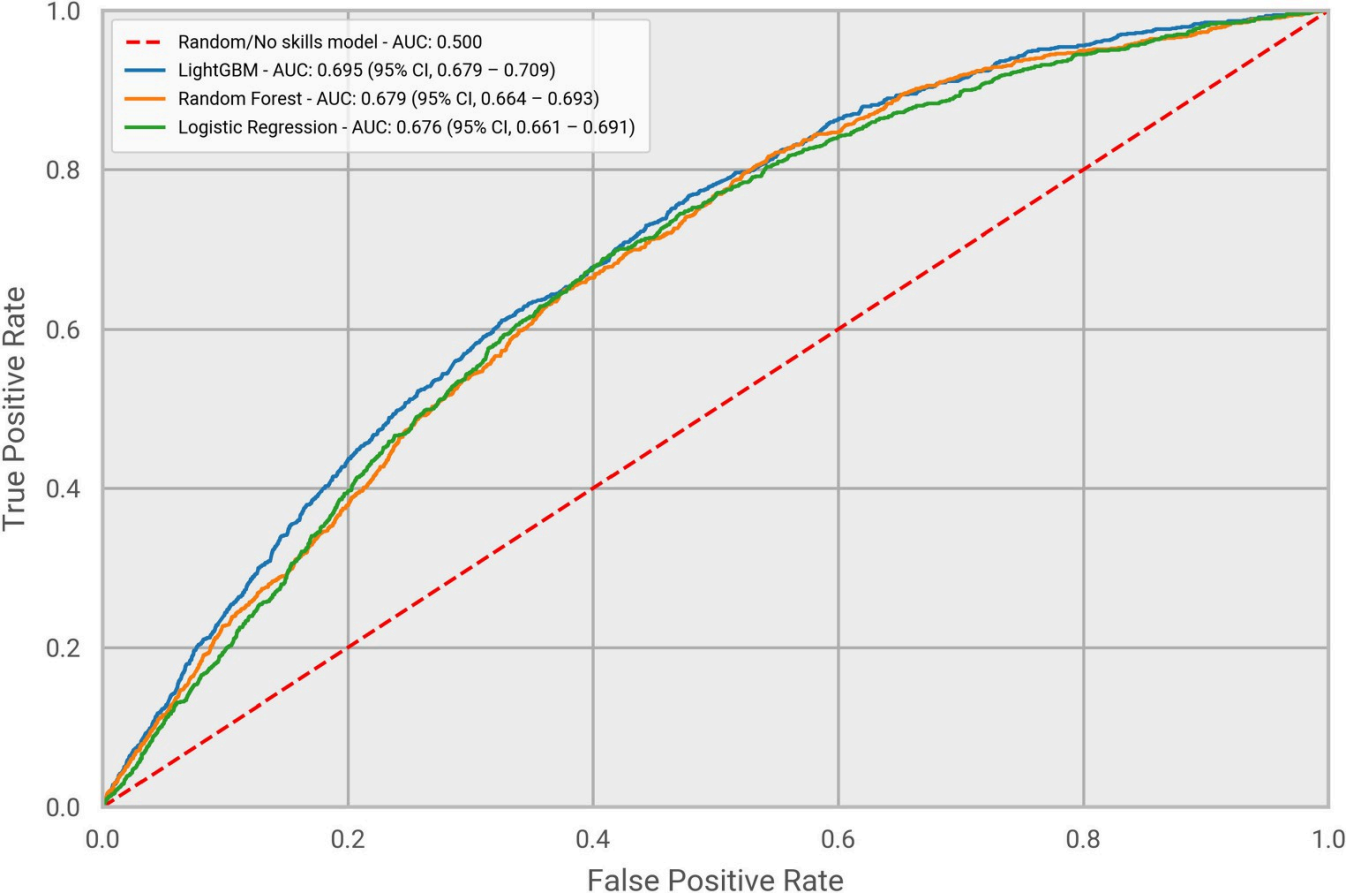
ROC curves. Performance evaluation of the three machine learning models, measured using a ROC curve.

**Fig 4:**
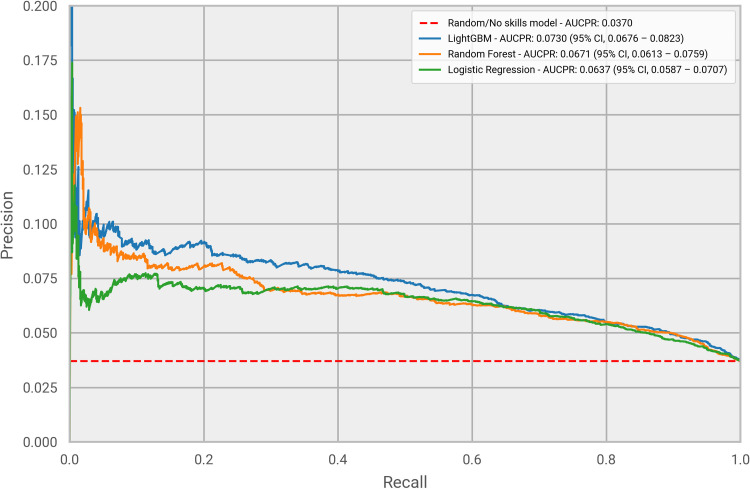
PR curves. Performance evaluation of the three machine learning models, measured using a Precision Recall curve.

Hyperopt was used to identify the hyper-parameters for each of the tested models. Those were selected as the parameters providing the highest performance on the training set, measured using a standard 5-fold cross-validation evaluation setting. Once the best parameters were found, the final models were built using the entire training set and their performance was evaluated on the test set (which was not used for the learning task). Figs 3 and 4 compare the performance of each model assessed using both a ROC curve and a PR curve. The LightGBM model was clearly the best performing under both evaluation metrics. This is not surprising as it has been extensively shown that GBDT outperforms other machine learning approaches, also in a healthcare setting [27]. As Fig 3 illustrates, the LightGBM model obtained an AUC of 0.695 (95% CI, 0.679–0.709), superior to the values of 0.679 (95% CI, 0.664–0.693) and 0.676 (95% CI, 0.661–0.691) from Random Forest and Logistic Regression, respectively. All the models had a performance significantly above the value of 0.5, which refers to a no-skills classifier, with the LightGBM model outperforming it by more than 40%. Similar results are illustrated in Fig 4 for Precision Recall curves. LightGBM was again the top performing model, with an AUCPR of 0.0730 (95% CI, 0.0676 – 0.0823), higher than the AUCPR obtained by the other two models, 0.0671 (95% CI, 0.0613–0.0759) and 0.0637 (95% CI, 0.0587 – 0.0707) respectively for Random Forest and Logistic Regression. The PR curve indicates that our model could extract meaningful patterns from the data that lead to a correct prediction of ARDS in patients from the test set. We found ARDS prevalence in our study to be lower than that in the data used by most of the published related work. This is explained by the fact that we did not only focus on a small cohort comprised of a subset of COVID-19 patients (e.g. hospitalized patients), but we included a wider range of patients diagnosed with COVID-19, as captured by IQVIA’s claims data. Nevertheless, we should note that for the performance evaluation of the models we did not re-balance the test set, and we have used the 1:26 positive to negative class ratio. This approach was adopted to avoid introducing bias in our metrics by having a test set with a distribution of patient types significantly different to that of real-world populations [28]. Given the higher performance of the LightGBM model, we decided to solely focus on it for the remaining of the analysis.

### Model interpretability

**Fig 5:**
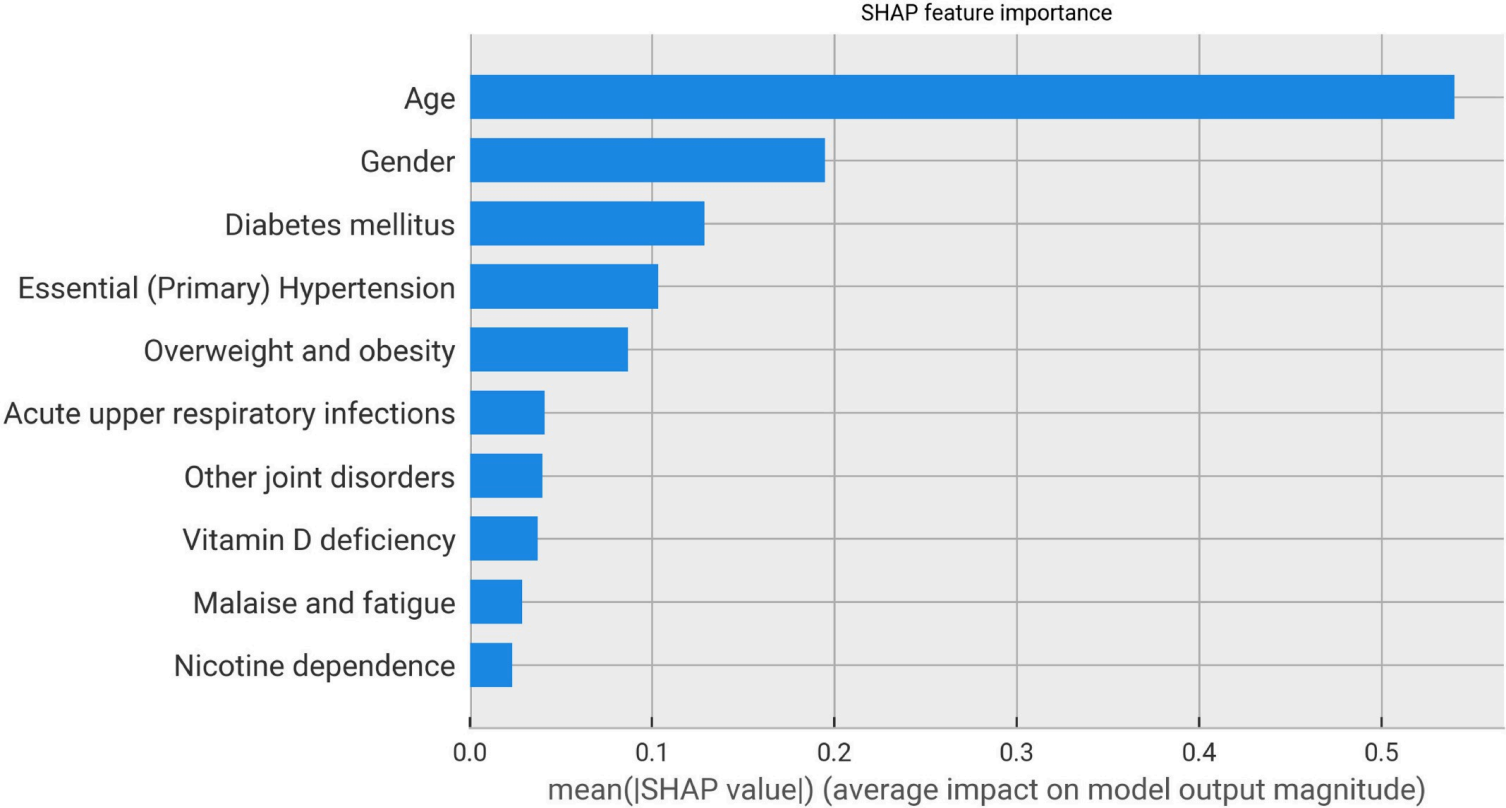
Most important predictive features. Top 10 features with highest absolute SHAP values.

We have applied the SHAP methodology to identify the most important features used by the LightGBM model to predict the presence of ARDS in COVID-19 patients. SHAP explains the contribution of each feature towards the prediction made by the model for a single patient. We have aggregated those importance values across the full cohort of patients, and we have plotted the most important features in Fig 5.

**Fig 6:**
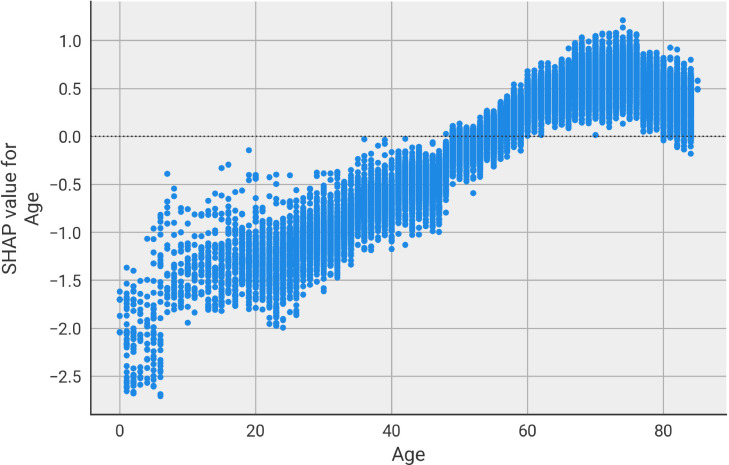
Age feature importance. Analysis of the importance of age towards prediction of ARDS in COVID-19 patients. Each dot represents a patient of the test set, indicating the SHAP value associated to their age.

The bar plot indicates age as the main feature used by the model to forecast the presence of ARDS. This is not surprising, as it is widely known that older patients are more likely to encounter severe complications from the infection. The distribution of SHAP values for the age feature (i.e. importance of age values for the prediction of the patients in the cohort) in Fig 6 reinforces the finding that older people are more likely to develop ARDS, according to the model. In the scatter plot each dot represents a patient from the cohort and its y-axis value shows the SHAP value associated to the patient’s age. Patients with age up to 50 are considered to have a smaller risk of ARDS (purely based on age contribution), as a negative SHAP value contribute towards the prediction of non-ARDS. Conversely, as age increases, its contribution towards the presence of ARDS increases as well, with has a small dip at the age of 80.

Gender was also relevant, with ARDS more present in Male patients as seen on Table 1. Diabetes mellitus, Hypertensive Diseases and Obesity were the top three most contributing comorbidities to model predictions. All of them have been confirmed to play a crucial role in the development of severe COVID-19 as reported in the medical literature. CDC has included them in the list of medical conditions that are more likely to lead to a severe infection [1]. In a Swedish nation-wide study, type 2 diabetes was independently associated with increased risk of hospitalization, admission to intensive care and death for COVID-19 patients [29]. Specialized literature has also shown how COVID-19 severity Is tripled in the diabetes community [30]. Hypertension seems also linked to a higher risk of COVID-19 severity, that might aggravate myocardial injury, including endothelial dysfunction, arterial stiffness, and left ventricle hypertrophy [31]. Additionally, obesity has been extensively proven to affect the severity of the infection, in studies conducted all around the world, from England [32] to United States [33] to China [34]. Finally, there is a link between nicotine addiction and COVID-19 severity, as reported in [35].

### Comparison to clinicians’ predictions

**Fig 7:**
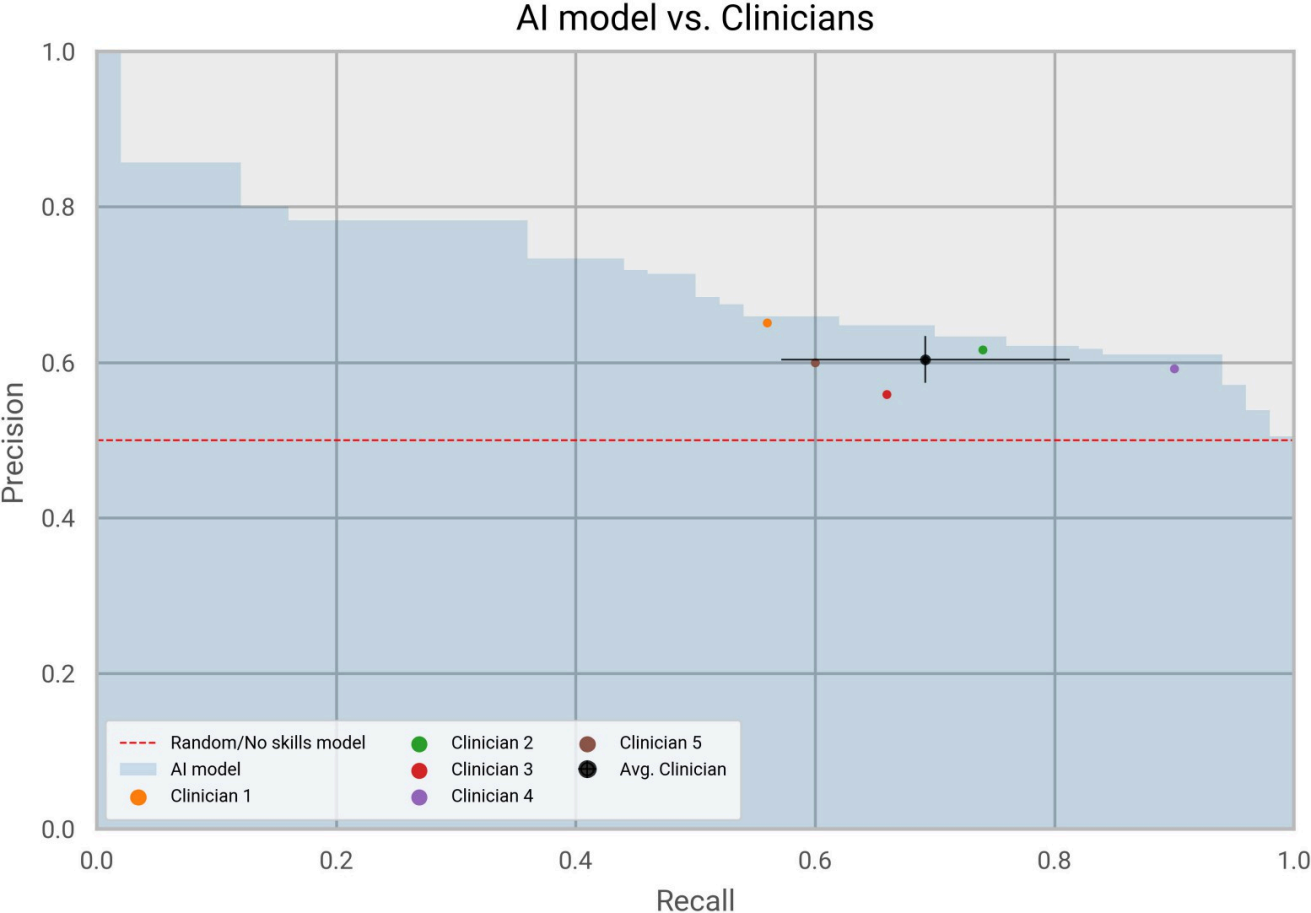
Machine learning vs. clinicians. Performance comparison between the machine learning model and five clinicians.

To further validate our predictive model, we compared its performance to that of individual clinicians, following best practices adopted by healthcare studies that employed Artificial Intelligence for disease prediction [36, 37]. We have asked a panel of five clinicians to predict who among 100 patients would develop ARDS. Patients were randomly selected from the 10% test set not used for training, 50 of them with ARDS and 50 without ARDS. Clinicians were provided with a list of comorbidities (i.e. diagnoses) for each of the patients and were asked to mark the patients that would have developed ARDS within four months from COVID-19 diagnosis. A new LightGBM model trained only on the top 15 most relevant features (with highest information gain from the original model) was also queried to predict the presence of ARDS in the same cohort. We focused on a reduced feature set to have a model that could realistically be used in a healthcare setting environment, where patients or practitioners could self-report the presence of only 15 comorbidities. The comparison of predictive performance between the model and the clinicians, measured using the PR curve, is provided in Fig 7. Clinicians showed a large variation in recall values, while obtaining similar precision. The ML model was on par or outperformed predictions made by the clinicians and exceeded the results of the “Average clinician”. The highest precision from a clinician was 65%, at 56% recall. At the same precision level, the model had 62% recall. Similarly, the highest recall obtained by a clinician was 90%, at 59% precision, while at the same recall level the model precision was 61%.

## Discussion

In this study we have developed a machine learning model to predict the development of ARDS in COVID-19 patients. We have trained and evaluated three different machine learning models and we identified LightGBM, a Gradient Boosted Decision Tree model, as the best performing approach. The analysis was performed using real-world data, based on US prescriptions and medical claims, that allowed us to create a large cohort of almost 290,000 patients diagnosed with COVID-19. Such an extensive dataset differentiates this work from most of the studies published so far on COVID-19 severity, often focused on smaller sets of patients. In addition, as claims data provide a very broad view of the patient clinical history, we were able to provide the model with more than 800 diagnosis codes, covering an extensive range of potential risk factors. Finally, IQVIA datasets capture patient level diagnosis and procedure information for more than 205 million non-identified patients, and they provide coverage for up to 92% of retail pharmacy prescriptions. This resulted in a dataset capturing 289,351 COVID-19 patients with a positive-to-negative ratio of 1:26. Consequently, we were able to evaluate the model performance on an experimental setting that more closely represents the distribution of ARDS in COVID-19 patients, and not under the simplifying assumption of a more balanced dataset.

The model performance was evaluated using 10% of unseen data coming from the original cohort, kept aside from training. ROC and PR curves showed a clear improvement in performance compared to a classifier prediction based only on the ARDS prevalence in our cohort (AUC of 0.695 vs. 0.500 and AUPRC of 0.073 vs. 0.037). This shows that meaningful patterns were discovered by LightGBM during training, that helped to correctly predict the presence of ARDS. The precision of 7.5% at 50% recall indicates that approximately 1 out of 13 patients predicted by the model as high-risk, will develop ARDS. This suggests that the model detects a population of patients with twice the cohort baseline risk. This precision is clinically useful in cases where the prevalence is very low, such as 3.6% in the case of our cohort. The ML model was further compared with the clinical judgment of five clinicians that were provided with the list of comorbidities affecting 100 COVID-19 patients and asked to identify who was more likely to develop ARDS. As reported in the Results, the model produced performance on par or better with the experts, showing equivalent precision and recall values with the best performing clinicians. A great advantage of Machine Learning models is their ability to scale to a large population, we could potentially screen an entire country population in matter of minutes, not possible with traditional healthcare settings. Overall, the comparison between the model and the experts suggests that our model could be potentially used in a healthcare setting to support clinicians in assessing the risk of ARDS in COVID-19 patients.

To identify which features contributed the most towards the prediction of ARDS, we have used SHAP, a game theory based approach for explaining machine learning model predictions. Age, together with gender, were the most contributing features. It is widely known that older people are more at risk of developing severe infections. The main comorbidities (namely diabetes, hypertension, and obesity) used by the model to decide on the presence of ARDS, have been extensively linked to severe COVID-19 in the literature. CDC has already added them to the list of medical conditions that can lead to a critical infection. It is important to stress that we have not performed any filtering or selection on the list of comorbidities to be used as input by the model. We have collected and used every ICD code present in the history of the patients, no a-priori hypothesis was used to bias the inclusion of information. The model automatically identified the most relevant features for the prediction of ARDS. Therefore, the validation of the top contributing features by independent studies as relevant for COVID-19 severity further highlights the value of the proposed approach.

The results presented in this study indicate that there is substantial benefit in applying ML models for predicting COVID-19 disease severity. However, there are some limitations to this work. First, the cohort used for modelling was built using claims data. Although claims data allowed for a large coverage of the population, they often under-represent patients that have a limited access to healthcare. This could potentially introduce a sample bias and could affect the performance of the model if deployed in real-world settings. Second, we have used “open” claims data, that is there is no minimum period a patient needs to be enrolled in claims to be selected in our cohort. Not setting any restriction has provided us with a bigger sample size, however this might have also included patients with partial or incomplete clinical history. Third, due to the nature of claims data, sometimes there might be a delay from when an event occurs to when it is registered as a claim and processed by the vendors. Therefore, to address this data caveat we decided to increase the outcome window used to identify severity. Nevertheless, this resulted in prevalence of ARDS which is in line with published literature at that time [38]. Fourth, we have used ARDS as proxy for COVID-19 severity. Despite ARDS being one of the most severe complications of COVID-19, especially for the initial *alpha* variant of the disease, inclusion of additional symptoms (e.g. Pneumonia or Acute Bronchitis) could result in a more detailed and precise positive cohort. This however would likely affect the size of the training data and could potentially lead to a less performing and reliable model. Fifth, validating the ML model’s performance against a higher number of clinical experts could further strengthen its credibility and could facilitate its adoption. Finally, this study would benefit from further external validation using a cohort based on a different population and if, available, different data sources.

Looking at directions of future research, we plan to use EMR data to assess how models trained on that data compare to this study. In addition, we also aim to retrain and evaluate the model after updating the patient cohort. The study focused on patients diagnosed with COVID-19 in April 2020. We are interested in extending the period in which we select COVID-19 patients to several months, to obtain a larger cohort and potentially help the algorithm to detect even more patterns. There also exist a few publicly available tools that either predict COVID-19 severity or mortality, such as the work published by Marcos et al. [9]. In the future, we aim to compare our updated model, trained on the most recent available data, with one or more predictive tools. Finally, by using a similar machine learning approach to study different time periods, we could better understand the impact of new COVID-19 variants, interrogate our data around vaccine efficacy, and subtle long-term side effects. We could even investigate optimal vaccination strategies by different patient segments (by age or comorbidity segments).

## Conclusion

Using longitudinal prescription data and office based medical claims data from over 289,000 U.S. patients, our gradient boosting decision trees model classified severe cases of COVID-19, with an AUC of 0.695 (95% CI, 0.679–0.709) and AUPRC of 0.0730 (95% CI, 0.0676 – 0.0823), outperforming two other machine learning algorithms. We also validated the model’s performance against human clinicians, with the results being on par or better both in terms of precision and recall. The most important predictors used by the model to classify severity, such as age, diabetes, and hypertension, agree with what has been observed in related studies. Our results indicate that the application of machine learning to claims datasets can contribute to the prediction of COVID-19 patients at risk of developing severe disease, which allows for better hospital resource allocation and patient prioritization.

## Supporting information

S1 File Input features. Complete list of ICD 10 codes used as input features to train the predictive models.
(CSV)

S2 File Performance confidence intervals. Confidence interval values calculated via bootstrap method for each of the tested classifiers.
(XLSX)

S1 Table List of hyperparameters. List of the hyperparameters evaluated by Hyperopt for each of the tested classifiers.
(DOCX)

## References

[1] CDC. People at increased risk of severe Covid-19. [Online].; 2021 [cited 2021. Available from: https://www.cdc.gov/coronavirus/2019-ncov/need-extra-precautions/people-with-medical-conditions.html.

[2] Sarke IH. Machine Learning: Algorithms, Real-World Applications and Research Directions. SN Computer Science. 2021. 10.1007/s42979-021-00592-x PMC7983091 33778771

[3] Zhanga Z, Chen L, Xu P, Yucai H. Predictive analytics with ensemble modeling in laparoscopic surgery: A technical note. Laparoscopic, Endoscopic and Robotic Surgery. 2022.

[4] Bohr A, Memarzadeh K. The rise of artificial intelligence in healthcare applications. 2020;: 25–60.

[5] Wong KCY, Xiang Y, So HC. Uncovering clinical risk factors and prediction of severe COVID-19: A machine learning approach based on UK Biobank data. Medrxiv. 2021. doi: 10.2196/29544 34591027 PMC8485986

[6] Dun C, Walsh CM, Bae S, Adalja A, Toner E, Lash TA, et al. A Machine Learning Study of 534,023 Medicare Beneficiaries with COVID-19: Implications for Personalized Risk Prediction. MedRxiv. 2020.

[7] Patel D, Kher V, Desai B, Lei X, Cen S, Nanda N, et al. Machine Learning Based Predictors for COVID-19 Disease Severity. Scientific Report. 2021. doi: 10.1038/s41598-021-83967-7 33633145 PMC7907061

[8] Fernandes FT, de Oliveira TA, Teixeira CE, de Moraes Batista AF, Costa GD, Filho ADPC. A multipurpose machine learning approach to predict COVID-19 negative prognosis in São Paulo, Brazil. Scientific Report. 2021; 11. doi: 10.1038/s41598-021-82885-y 33558602 PMC7870665

[9] Marcos M, Belhassen-García M, Sánchez-Puente A, Sampedro-Gomez J, Azibeiro R, Dorado-Díaz PI, et al. Development of a severity of disease score and classification model by machine learning for hospitalized COVID-19 patients. PlosOne. 2021. doi: 10.1371/journal.pone.0240200 33882060 PMC8059804

[10] Cheng FY, Joshi H, Tandon P, Freeman R, Reich DL, Mazumdar M, et al. Using Machine Learning to Predict ICU Transfer in Hospitalized COVID-19 Patients. Journal of Clinical Medicine. 2020; 9: 1668. doi: 10.3390/jcm9061668 32492874 PMC7356638

[11] Ferrari D, Milic J, Tonelli R, Ghinelli F, Meschiari M, Volpi S, et al. Machine learning in predicting respiratory failure in patients with COVID-19 pneumonia—Challenges, strengths, and opportunities in a global health emergency. Plos One. 2020. 10.1371/journal.pone.0239172 PMC7660476 33180787

[12] Xu W, Sun NN, Gao HN, Chen ZY, Yang Y, Ju B, et al. Risk Factors Analysis of COVID-19 Patients with ARDS and Prediction Based on Machine Learning. Scientific Report. 2021. doi: 10.1038/s41598-021-82492-x 33536460 PMC7858607

[13] DeCaprio D, Gartner J, McCall CJ, Burgess T, Garcia K, Kothari S, et al. Building a COVID-19 vulnerability index. Journal of Medical Artificial Intelligence. 2020; 3: 15–15.

[14] Jain A, Chaurasia R, Sengar NS, Singh M, Mahor S, Narain S. Analysis of vitamin D level among asymptomatic and critically ill COVID-19 patients and its correlation with inflammatory markers. Scientific reports. 2020; 10: 1–8. 33214648 10.1038/s41598-020-77093-z PMC7677378

[15] Singhal L, Garg Y, Yang P, Tabaie A, Wong AI, Mohammed A, et al. eARDS: A multi-center validation of an interpretable machine learning algorithm of early onset Acute Respiratory Distress Syndrome (ARDS) among critically ill adults with COVID-19. Plos One. 2021. doi: 10.1371/journal.pone.0257056 34559819 PMC8462682

[16] Singh V, Kamaleswaran R, Chalfin D, Bu, San Roman J, Rojas-Kenney E, et al. A deep learning approach for predicting severity of COVID-19 patients using a parsimonious set of laboratory markers. iScience. 2021. doi: 10.1016/j.isci.2021.103523 34870131 PMC8626152

[17] Yibai X, Yan M, Lianguo R, Dan L, Cheng L, Luqi H, et al. Comparing different machine learning techniques for predicting COVID-19 severity. Infectious Diseases of Poverty. 2022. 10.1186/s40249-022-00946-4 PMC8851750 35177120

[18] Wright R. Logistic regression. 1995.

[19] Breiman L. Random Forests. 2001.

[20] Ke G, Meng Q, Finley T, Wang T, Chen W, Ma W, et al. LightGBM: A Highly Efficient Gradient Boosting Decision Tree. In Proceedings of the 31st International Conference on Neural Information Processing Systems; 2017; Red Hook, NY, USA: Curran Associates Inc. p. 3149–3157.

[21] Bergstra J, Bardenet R, Bengio Y, Kégl B. Algorithms for Hyper-Parameter Optimization. In Proceedings of the 24th International Conference on Neural Information Processing Systems; 2011; Red Hook, NY, USA: Curran Associates Inc. p. 2546–2554.

[22] Zhu M. Recall, precision and average precision.; 2004.

[23] Takaya Saito MR. The precision-recall plot is more informative than the ROC plot when evaluating binary classifiers on imbalanced datasets. Plos One. 2015. 10.1371/journal.pone.0118432 PMC4349800 25738806

[24] Lundberg S, Lee SI. A unified approach to interpreting model predictions. CoRR. 2017; abs/1705.07874.

[25] Lundberg SM, Erion GG, Chen H, DeGrave A, Prutkin JM, Nair B, et al. Explainable AI for Trees: From Local Explanations to Global Understanding. CoRR. 2019; abs/1905.04610. 10.1038/s42256-019-0138-9 PMC7326367 32607472

[26] Lundberg SM, Nair B, Vavilala MS, Horibe M, Eisses MJ, Adams T, et al. Explainable machine learning predictions to help anesthesiologists prevent hypoxemia during surgery. bioRxiv. 2017. 10.1038/s41551-018-0304-0 PMC6467492 31001455

[27] Olson RS, Cava WL, Mustahsan Z, Varik A, Moore JH. Data-driven Advice for Applying Machine Learning to Bioinformatics Problems. 2018. PMC5890912 29218881

[28] Chen C, Liu Y, Peng L. How to Develop Machine Learning Models for Healthcare. Nature Materials. 2019. doi: 10.1038/s41563-019-0345-0 31000806

[29] Rawshani A, Kjölhede EA, Rawshani A, Sattar N, Eeg-Olofsson K, Adiels M, et al. Severe COVID-19 in people with type 1 and type 2 diabetes in Sweden: A nationwide retrospective cohort study. The Lancet Regional Health-Europe. 2021; 4: 100105. doi: 10.1016/j.lanepe.2021.100105 33969336 PMC8086507

[30] Gregory JM, Slaughter JC, Duffus SH, Smith TJ, LeStourgeon LM, Jaser SS, et al. COVID-19 severity is tripled in the diabetes community: a prospective analysis of the pandemic’s impact in type 1 and type 2 diabetes. Diabetes Care. 2021; 44: 526–532. doi: 10.2337/dc20-2260 33268335 PMC7818316

[31] Tavares CAM, Bailey MA, Girardi ACC. Biological context linking hypertension and higher risk for COVID-19 severity. Frontiers in physiology. 2020; 11: 1510. doi: 10.3389/fphys.2020.599729 33329052 PMC7710931

[32] Gao M, Piernas C, Astbury NM, Hippisley-Cox J, O’Rahilly S, Aveyard P, et al. Associations between body-mass index and COVID-19 severity in 6.9 million people in England: a prospective, community-based, cohort study. The Lancet Diabetes & Endocrinology. 2021; 9: 350–359. doi: 10.1016/S2213-8587(21)00089-9 33932335 PMC8081400

[33] Kuehn BM. More Severe Obesity Leads to More Severe COVID-19 in Study. JAMA. 2021; 325: 1603–1603. doi: 10.1001/jama.2021.4853 33904860

[34] Gao F, Zheng KI, Wang XB, Sun QF, Pan KH, Wang TY, et al. Obesity is a risk factor for greater COVID-19 severity. Diabetes care. 2020; 43: e72–e74. doi: 10.2337/dc20-0682 32409499

[35] Gülsen A, Yigitbas BA, Uslu B, Drömann D, Kilinc O. The effect of smoking on COVID-19 symptom severity: systematic review and meta-analysis. Pulmonary medicine. 2020; 2020. doi: 10.1155/2020/7590207 32963831 PMC7499286

[36] Shen J, Zhang CJP, Jiang B, Chen J, Song J, Liu Z, et al. Artificial intelligence versus clinicians in disease diagnosis: systematic review. JMIR medical informatics. 2019; 7: e10010. doi: 10.2196/10010 31420959 PMC6716335

[37] Long E, Lin H, Liu Z, Wu X, Wang L, Jiang J, et al. An artificial intelligence platform for the multihospital collaborative management of congenital cataracts. Nature biomedical engineering. 2017; 1: 1–8.

[38] Matthay M, Leligdowicz A, Liu K. Biological Mechanisms of COVID-19 Acute Respiratory Distress Syndrome. American Journal of Respiratory and Critical Care Medicine. 2020. doi: 10.1164/rccm.202009-3629ED 32997945 PMC7706166

