# Supplementary material for: A machine learning model on Real World Data for predicting progression to Acute Respiratory Distress Syndrome (ARDS) among COVID-19 patients
Source: PLoS One. 2022 Jul 28;17(7):e0271227. doi: 10.1371/journal.pone.0271227 (PMC9333235; doi:10.1371/journal.pone.0271227)
Supplement: S1 Table — List of the hyperparameters evaluated by Hyperopt for each of the tested classifiers. (DOCX) [file pone.0271227.s003.docx]

**S2 Table. List of hyperparameters of each classifier evaluated by Hyperopt**

|  | **Hyperparameter** |
| --- | --- |
| **Logistic Regression** | Penalty |
|  | L1 ratio |
|  | C |
|  | Max iterations |
|  | estimators |
| **Random Forest** | Num estimators |
|  | Max Depth |
|  | Min samples leaf |
|  | Min samples split |
|  | Max features |
|  | Bootstrap |
| **LightGBM** | Num leaves |
|  | Num iterations |
|  | Min data leaf |
|  | Learning rate |
|  | Max bin |
|  | Max depth |

**LightGBM**: <https://lightgbm.readthedocs.io/> (version 2.2.3)

**Logistic Regression / Random Forest**: <https://scikit-learn.org/> (version 0.21.2)
